# The genome of *Medicago polymorpha* provides insights into its edibility and nutritional value as a vegetable and forage legume

**DOI:** 10.1038/s41438-021-00483-5

**Published:** 2021-03-01

**Authors:** Jiawen Cui, Zhaogeng Lu, Tianyi Wang, Gang Chen, Salma Mostafa, Hailong Ren, Sian Liu, Chunxiang Fu, Li Wang, Yingfang Zhu, Jinkai Lu, Xiang Chen, Zhenwu Wei, Biao Jin

**Affiliations:** 1 College of Horticulture and Plant Protection, Yangzhou University, 225009 Yangzhou, China; 2 College of Animal Science and Technology, Yangzhou University, 225009 Yangzhou, China; 3 Novogene Bioinformatics Institute, 100083 Beijing, China; 4 College of Bioscience and Biotechnology, Yangzhou University, 225009 Yangzhou, China; 5 Qingdao Institute of Bioenergy and Bioprocess Technology, Chinese Academy of Sciences, 266101 Qingdao, China; 6 State Key Laboratory of Crop Stress Adaptation and Improvement, Key Laboratory of Cotton Biology, School of Life Sciences, Henan University, 475001 Kaifeng, China

**Keywords:** Plant molecular biology, Genome

## Abstract

*Medicago polymorpha* is a nutritious and palatable forage and vegetable plant that also fixes nitrogen. Here, we reveal the chromosome-scale genome sequence of *M. polymorpha* using an integrated approach including Illumina, PacBio and Hi-C technologies. We combined PacBio full-length RNA-seq, metabolomic analysis, structural anatomy analysis and related physiological indexes to elucidate the important agronomic traits of *M. polymorpha* for forage and vegetable usage. The assembled *M. polymorpha* genome consisted of 457.53 Mb with a long scaffold N50 of 57.72 Mb, and 92.92% (441.83 Mb) of the assembly was assigned to seven pseudochromosomes. Comparative genomic analysis revealed that expansion and contraction of the photosynthesis and lignin biosynthetic gene families, respectively, led to enhancement of nutritious compounds and reduced lignin biosynthesis in *M. polymorpha*. In addition, we found that several positively selected nitrogen metabolism-related genes were responsible for crude protein biosynthesis. Notably, the metabolomic results revealed that a large number of flavonoids, vitamins, alkaloids, and terpenoids were enriched in *M. polymorpha*. These results imply that the decreased lignin content but relatively high nutrient content of *M. polymorpha* enhance its edibility and nutritional value as a forage and vegetable. Our genomic data provide a genetic basis that will accelerate functional genomic and breeding research on *M. polymorpha* as well as other *Medicago* and legume plants.

## Introduction

Legumes (Fabaceae or Leguminosae), the second most important group of crop plants, contribute an important portion of the plant protein and oil content in human and animal diets. Numerous efforts have been made to improve legume production and quality through various approaches, including breeding, agronomic practices, and genetics. Because genomic information is essential to crop improvement programs, multiple assembled genomes for legumes have been made available, including those for wild legume (*Lotus japonicus*)^1^, soybean (*Glycine max*)^2^, *Glycine latifolia*^3^, *Medicago* (*Medicago truncatula* and *Medicago sativa*)^4–7^, chickpea (*Cicer arietinum*)^8^, mungbean (*Vigna radiata*)^9^, and peanut (*Arachis duranensis*)^10^. Among species of *Medicago*, the genome of a model legume species (*M. truncatula*) was the first to be sequenced^4^ and updated^5^. More recently, the genome of alfalfa (*M. sativa*), known as the king of forage, has also been sequenced^6,7^. These genomes provide valuable resources for studying legume genomics as well as for crop breeding and improvement.

Legumes are important forage crops in livestock agriculture^3,7^. The nutritive value of forage can be assessed based on several key parameters, including protein content, total fiber concentration, leaf-to-stem ratio, and digestibility^11^. In particular, the concentration of lignin is negatively correlated with forage digestibility for ruminant animals^12^. Lignin is derived from the dehydrogenative polymerization of three different hydroxyl-cinnamyl alcohols (*p*-coumaryl alcohol, conifer alcohol and sinapyl alcohol). These substances give rise to units of the lignin polymer, including *p*-hydroxyphenyl (H), guaiacyl (G), and syringyl (S)^13^. In the forage industry, a small reduction in lignin content can improve digestibility for livestock, thereby benefiting farmers or ranchers^14^. In addition, forage is valued for high protein concentrations, as a high protein content can reduce the cost of protein supplementation in farm feeds^11^.

*Medicago polymorpha* L. (bur clover, 金花菜 jīn huā cài, 南苜蓿 nán mùxu, 秧草 yāng cǎo), is a relative of the legume model species *M. truncatula* and alfalfa (*M. sativa*). It is native to the Mediterranean Basin but is widespread around the world^15^. *M. polymorpha* is recognized as a nutritious and palatable forage plant that also fixes nitrogen^16,17^. Due to its high nutritional content and edibility, it is consumed both cooked and fresh in China. In recent years, *M. polymorpha* has been increasingly used for both animal feed and human consumption^18^. In addition to its economic value, *M. polymorpha* is a critical ecological plant in various farming systems worldwide, as it is a highly effective nitrogen fixer and organic soil improver that can be readily reseeded^19^. Despite its importance, information about the genome of *M. polymorpha* is still lacking. In this study, we constructed a high-quality genome of *M. polymorpha* at the chromosomal scale. Given its short lifespan and easy cultivation, *M. polymorpha* can be used as a model plant in the legume family for many potential applications. Moreover, the *M. polymorpha* genome data produced in this study will provide a valuable genetic resource for fundamental research and breeding programs related to other economically important *Medicago* plants.

## Results

### Genome sequencing, assembly, and annotation

**Fig. 1:**
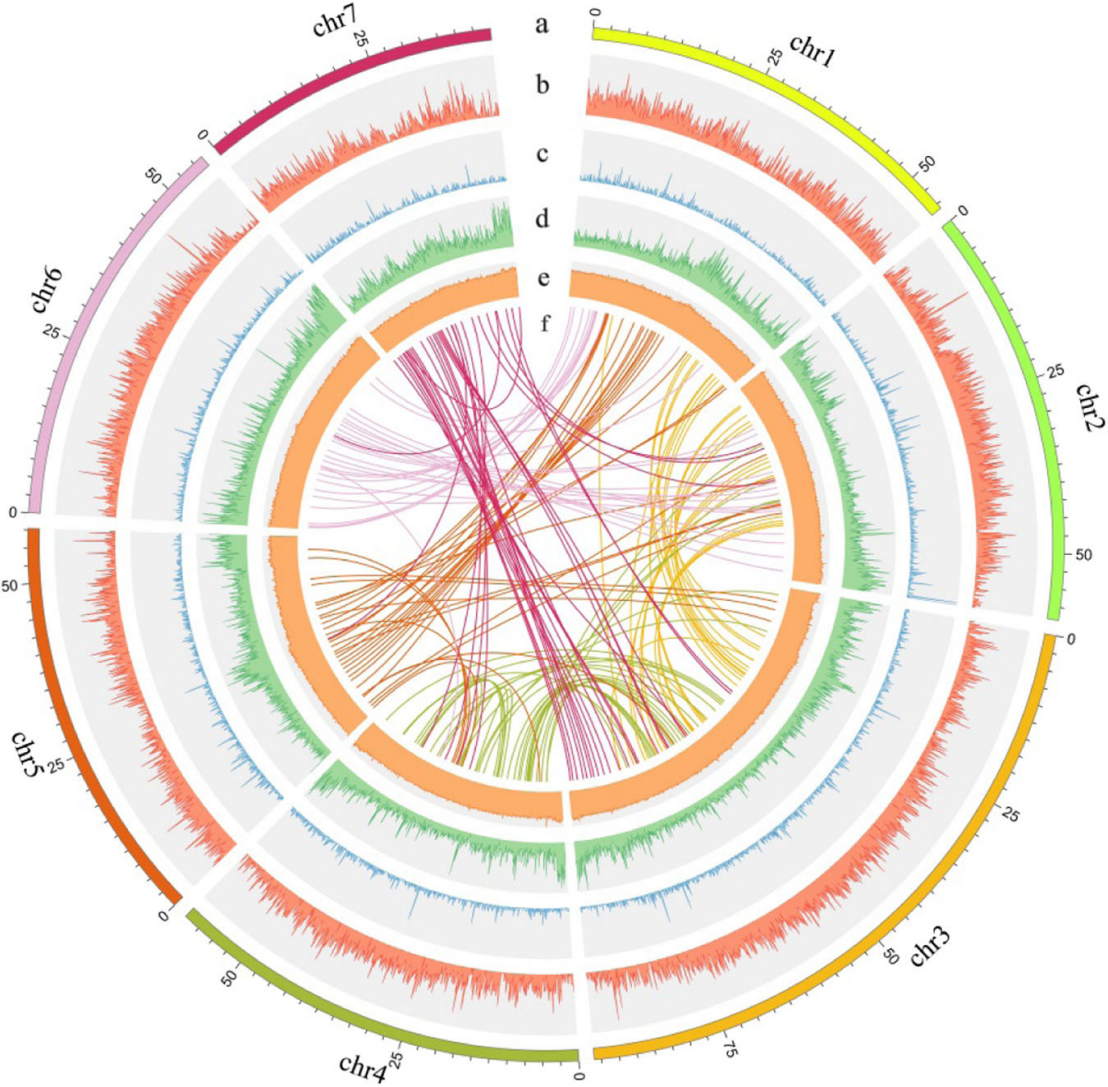
Genomic features of *M. polymorpha*. **a** Circular representation of the seven pseudochromosomes. **b** Density of genes. **c** Density of noncoding RNA. **d** Distribution of transposable elements (TEs). **e** Distribution of GC content. **f** Syntenic blocks within the genome of *M. polymorpha*. The different colored lines represent gene collinear connections between each chromosome and itself or other chromosomes (each line in collinearity indicates the syntenic gene)

We sequenced the genome of *M. polymorpha* ‘Huaiyang Jinhuacai’ (the main nationally authorized cultivar) using the Illumina and PacBio RSII platforms with assisted assembly correction conducted by chromatin conformation capture technology (Hi-C). A total of 59.2 Gb of Illumina short reads and 57.0 Gb of PacBio long reads were obtained, with approximately 112.83-fold and 117.18-fold high-quality sequence coverage of 505.19 Mb (size estimated by k-mer analysis, Table S1). K-mer analysis of the short reads also showed a low heterozygosity level of the *M. polymorpha* genome (Fig. S1). To assist the assembly correction, 123.89× Hi-C data (62.59 Gb) was used, and consequently, a contiguous reference genome of 457.53 Mb was generated with a contig N50 of 11.02 Mb and scaffold N50 of 57.72 Mb (Fig. 1 and Tables 1 and S1). Notably, the vast majority of core eukaryotic genes (96.77%) and genes in the Benchmarking Universal Single-Copy Orthologs (BUSCO) datasets (91.8%) were identified (Table S2). In addition, 92.92% of the genomic sequences were anchored onto seven pseudochromosomes (Table 1). A heatmap generated from Hi-C data suggests that all data bins could be clearly divided into seven pseudochromosomes (Figs. 1 and S2). Together, these analytical and statistical results for gene quality verified that our chromosomal-scale genome assembly was precise and complete.

**Table 1:** Assembly statistics for the *M. polymorpha* genome

Assembly features	Number	Length (bp)
Assembled genome size	–	457,531,409
N50 contig	14	11,017,972
N50 scaffold	4	57,718,482
N90 scaffold	7	42,472,796
Max. scaffold	–	93,525,394
Anchored and oriented scaffolds	73	441,829,113 (92.92%)

Annotation features		
No. of predicted protein-coding genes	36,087	
Average gene length (bp)	3358.49	
Average exons per gene	4.53	
miRNAs	2307	
tRNAs	817	
rRNAs	2281	
snRNAs	1334	
Mask repeat sequence length (Mb)	166.62	
Percentage of repeat sequences (%)	38.04	

To assist in annotating our genome, we generated RNA sequencing data from six libraries representing the main tissue types, namely, seeds, roots, stems, leaves, flowers, and pods. Moreover, we constructed a mixed library from all six tissues and sequenced the full-length transcriptome using the PacBio platform. After de novo screening, homology searching, transcript mapping (Fig. S3), and further filtering, we predicted a total of 36,087 protein-coding genes (Tables S3), 99.0% of which were functionally annotated (Table S4). We subsequently performed homology searches and annotated noncoding RNA genes, yielding 2307 miRNAs, 817 transfer RNAs, 2281 ribosomal RNAs, and 1334 small nuclear RNAs. Furthermore, we identified 166.62 Mb of repetitive sequences (accounting for 38.04% of the assembled genome) in the genome of *M. polymorpha* (Table 1).

### Comparative genomic analysis of *M. polymorpha*

**Fig. 2:**
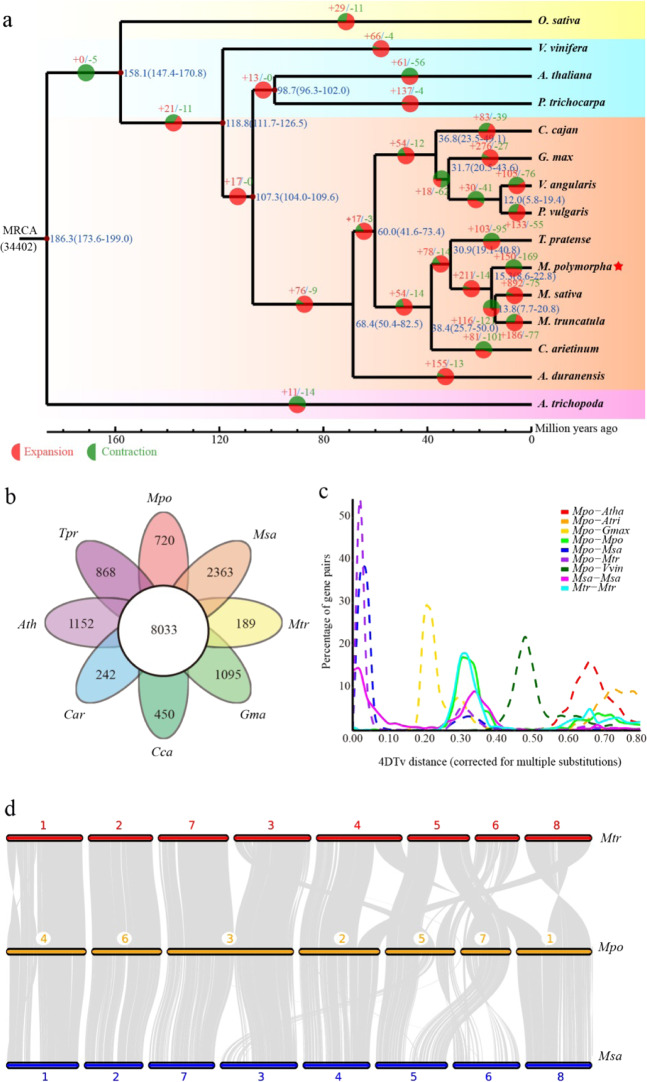
Comparative genomic analysis of the *M. polymorpha* genome. **a** Phylogenetic tree of 14 species, showing gene family expansion and contraction relative to the most recent common ancestor. The numeric value beside each node indicates the estimated divergence time of each node (blue) and the number of expanded (red) and contracted (green) gene families. **b** Venn diagram showing the shared and unique gene families among *M. polymorpha* and seven other plant species (*Mpo*, *M. polymorpha*; *Msa*, *M. sativa*; *Mtr*, *M. truncatula*; *Ath*, *A. thaliana*; *Car*, *C. arietinum*; *Cca*, *C. cajan*; *Tpr*, *T. pratense*). **c** 4DTv analysis. The *x*-axis represents the 4DTv value, and the y-axis represents the proportion of genes corresponding to each 4DTv value. **d** Synteny analysis of *M. polymorpha*, *M. truncatula*, and *M. sativa*

Next, we examined the evolutionary relationships among *M. polymorpha* and other sequenced plant genomes, including representatives of basal angiosperms (*Amborella trichopoda*), monocotyledons (*Oryza sativa*), dicotyledons (*Vitis vinifera*, *Arabidopsis thaliana*, *Populus trichocarpa*), and nine legumes. Phylogenetic analysis based on 328 single-copy gene families from 15 sequenced plant genomes supported a close relationship among *M. polymorpha*, *M. sativa* and *M. truncatula* in the *Medicago* genus. The phylogeny also reflected the divergence between *M. polymorpha* and the common ancestor of *M. truncatula* and *M. sativa*, which occurred ~15.3 million years ago (Mya) (Fig. 2a). In flowering plants, the expansion or contraction of gene families is an important driver of lineage differentiation and phenotypic diversification. Our results showed that 150 families comprising 1103 genes exhibited significant expansion in the *M. polymorpha* genome (Table S5). Functional annotation of these genes demonstrated that they were mainly enriched in functional categories involved in photosynthesis and spliceosomes (Fig. S4). Furthermore, 169 families, comprising 698 genes, exhibited significant contraction in the *M. polymorpha* genome (Table S6), and these genes were significantly enriched in functions related to the biosynthesis of secondary metabolites and limonene and pinene degradation (Fig. S5).

In addition, all protein-coding genes from eight genomes (*M. polymorpha*, *M. truncatula*, *M. sativa*, *G. max*, *C. arietinum*, *Cajanus. cajan*, *T. pratense*, and *A. thaliana*) were clustered into 8033 common gene families (Fig. 2b), and 720 gene families were unique to *M. polymorpha*. Functional enrichment analysis of *M. polymorpha*-specific genes based on both Gene Ontology (GO) and the Kyoto Encyclopedia of Genes and Genomes (KEGG) revealed that these gene families were significantly enriched in genes related to vitamin B6 metabolism, histidine metabolism and response to stimulus (Fig. S6).

We used MCscanX to identify synteny blocks within the genomes of *M. polymorpha* and related species. Based on the abundance of 4DTv sites, one significant peak was found in the *M. polymorpha*, *M. truncatula* and *M. sativa* genomes (the green peak of *Mpo*-*Mpo*, 4DTv: 0.30) (Fig. 2c; Table S7), indicating that the whole-genome duplication (WGD) event (~38.00–70.37 Mya, Fig. S7) occurred before the divergence of *G*. *max* and *Medicago*, supporting the ancestral Papilionoideae WGD event.

Synteny block analysis is often used to determine chromosome evolution among related species. Here, we analyzed the aligned protein sequences of *M. polymorpha* in comparison with both *M. truncatula* and *M. sativa*. The results showed strong genomic syntenic relationships among *M. polymorpha*, *M. truncatula* and *M. sativa* (Fig. 2d). A chromosomal fusion event observed in the synteny results indicates that chromosome 3 (chr 3) of *M. polymorpha* arose from the fusion of chr 3 and chr 7 of *M. truncatula* and *M. sativa* (Fig. 2d).

### Genetic basis of lignin biosynthesis in *M. polymorpha*

**Fig. 3:**
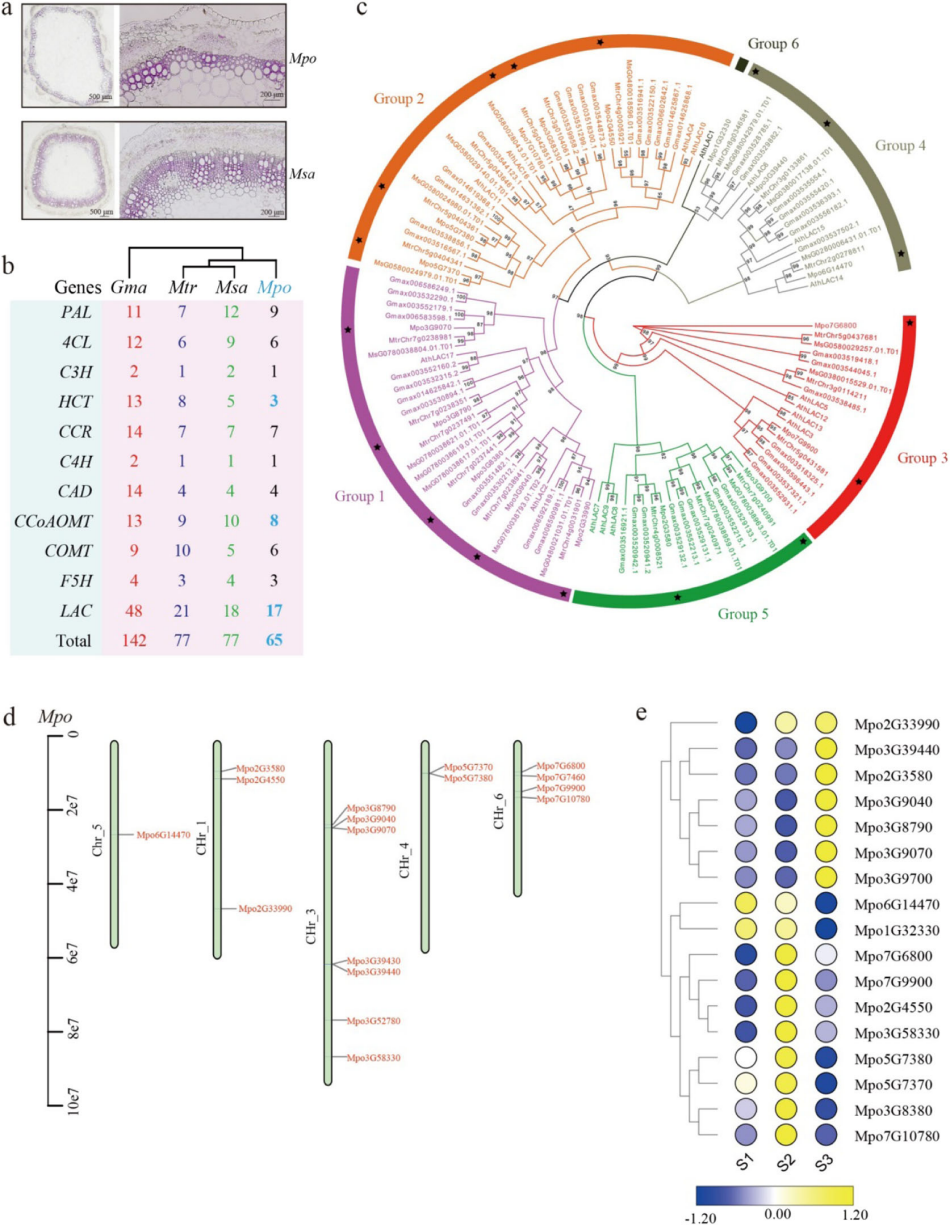
Analysis of lignin-biosynthesis-related genes. **a** Anatomical specimens of xylem from *M. polymorpha* (*Mpo*) and *M. sativa* (*Msa*). **b** Copy numbers of genes involved in the lignin biosynthetic pathway in *M. polymorpha*, *M. truncatula*m, *M. sativa* and *G. max*. PAL phenylalanine ammonia-lyase, 4CL 4-coumarate:CoA ligase, C3H p-coumarate 3-hydroxylase, HCT hydroxycinnamoyl-CoA:shikimate hydroxycinnamoyl transferase, CCR cinnamoyl-CoA reductase, C4H cinnamate 4-hydroxylase, CAD cinnamyl alcohol dehydrogenase, CCoAOMT caffeoyl-CoA O-methyltransferase, COMT caffeic acid O-methyltransferase, F5H ferulate 5-hydroxylase, LAC laccase. **c** Phylogenetic relationships of all laccases from *A. thaliana, M. polymorpha*, *M. truncatula*, *M. sativa*, and *G. max*. The laccase sequences from *A. thaliana*, *G. max*, *M. sativa*, *M. truncatula*, and *M. polymorpha* were aligned by Clustal X, and the phylogenetic tree was constructed using IQ-TREE by the maximum likelihood method. The displayed bootstrap values are based on 1000 replicates. The laccase family members were classified into six groups, which are denoted by differently colored branches. **d** Distribution of laccases along *M. polymorpha* chromosomes. **e** Expression levels of laccase genes at three growth stages. The expression values of laccase genes at the row scale were normalized, and the values are indicated by a continuous color scheme. Yellow indicates a high expression level, and blue indicates a low expression level

Lignins are phenylpropanoid-derived polymers generated from the oxidative polymerization of three major hydroxycinnamyl alcohols. We compared stem structure differences between *M. polymorpha* and *M. sativa* using paraffin sections. The sections showed that cell layers in the xylem of *M. sativa* were thicker than those in *M. polymorpha*, indicating a lower lignin content in *M. polymorpha* stems (Fig. 3a). Furthermore, using the Basic Local Alignment Search Tool (BLAST), a conserved domain search and manual curation tool, we identified orthologs of genes involved in lignin biosynthesis (Table S8). We found that 65 lignin-biosynthesis-related genes are present in the *M. polymorpha* genome, while 142 are present in *G. max*, 77 in *M. truncatula* and 77 in *M*. *sativa*. The numbers of genes encoding hydroxycinnamoyl-CoA:shikimate hydroxycinnamoyl transferase (HCT), caffeoyl-CoA O-methyltransferase (CCoAOMT), caffeic acid O-methyltransferase (COMT), and particularly laccase were significantly lower than those in the other species tested (Fig. 3b). Laccase is reportedly involved in the polymerization of lignin precursors^13^. We identified a total of 17 laccases in the *M. polymorpha* genome. We further constructed a molecular phylogenetic tree of laccases from *M. polymorpha*, *M. sativa*, *A. thaliana*, *G. max*, and *M. truncatula* using IQ-TREE software. The phylogenetic tree indicated that *M. polymorpha* laccase genes are clustered into five groups (Groups 1–5). Five members (*Mpo3G9040*, *Mpo3G8380*, *Mpo3G8790*, *Mpo2G33990*, and *Mpo3G9070*) were placed in Group 1, five members (*Mpo2G4550*, *Mpo7G10780*, *Mpo5G7380*, *Mpo5G7370*, and *Mpo3G58330*) in Group 2, two members (*Mpo7G6800* and *Mpo7G9900*) in Group 3, three members (*Mpo6G14470*, *Mpo3G39440*, and *Mpo1G32330*) in Group 4 and two members (*Mpo3G9700* and *Mpo2G3580*) in Group 5 (Fig. 3c). These genes were distributed on chromosomes 1, 3, 4, 5, and 6 (Fig. 3d). Expression analysis indicated that 7 of these genes were highly expressed in S3, and 10 were expressed significantly in S2 (S1: seedling stage; S2: early flowering stage; S3: late flowering stage) (Fig. 3e).

**Fig. 4:**
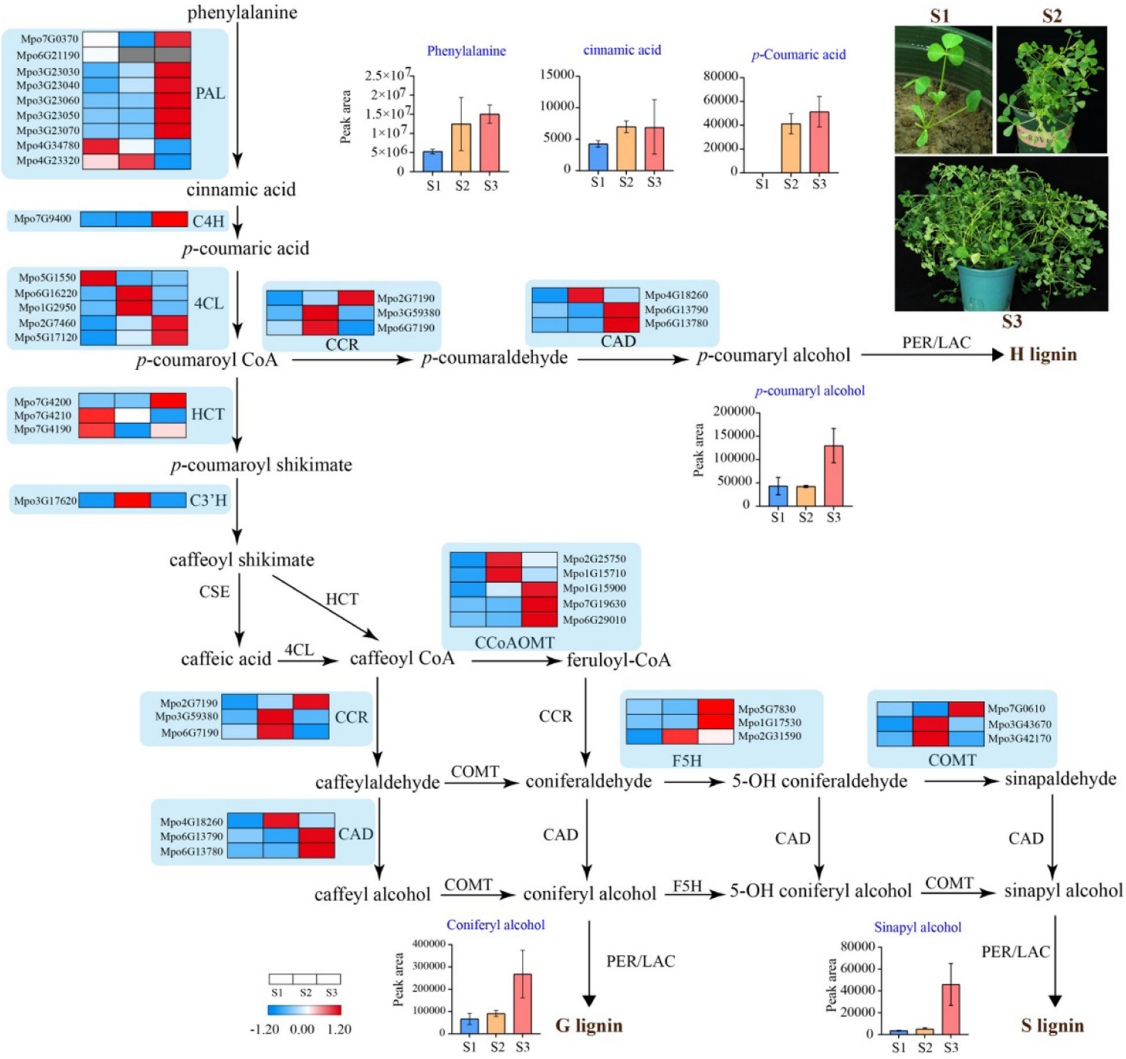
Diagram of the lignin biosynthetic pathway. Heatmaps showing the expression levels of lignin-biosynthesis-related genes at various growth stages. The expression values (FPKM) at the row scale were normalized. Higher mRNA expression levels are indicated by red, and lower mRNA expression levels are indicated by blue, whereas nonexpressed mRNAs are shown in gray; column diagrams show the levels of metabolites in the lignin biosynthetic pathway

Moreover, we examined the expression of lignin-biosynthesis-related genes from different growth stages using RNA-seq. Using transcriptome data, we identified 36 lignin-biosynthesis-related genes, of which 19 and 11 were upregulated at S3 and S2, respectively. This finding is consistent with our metabolomic results showing that the levels of several metabolites in the biosynthesis pathway of lignin, in particular *p*-coumaryl, coniferyl, and sinapyl alcohol, increased significantly from S1 and S2 to S3 (Fig. 4). In addition, the content of sinapyl alcohol was lower than that of coniferyl alcohol at S2.

### Detection of nutritional substances in *M. polymorpha*

**Fig. 5:**
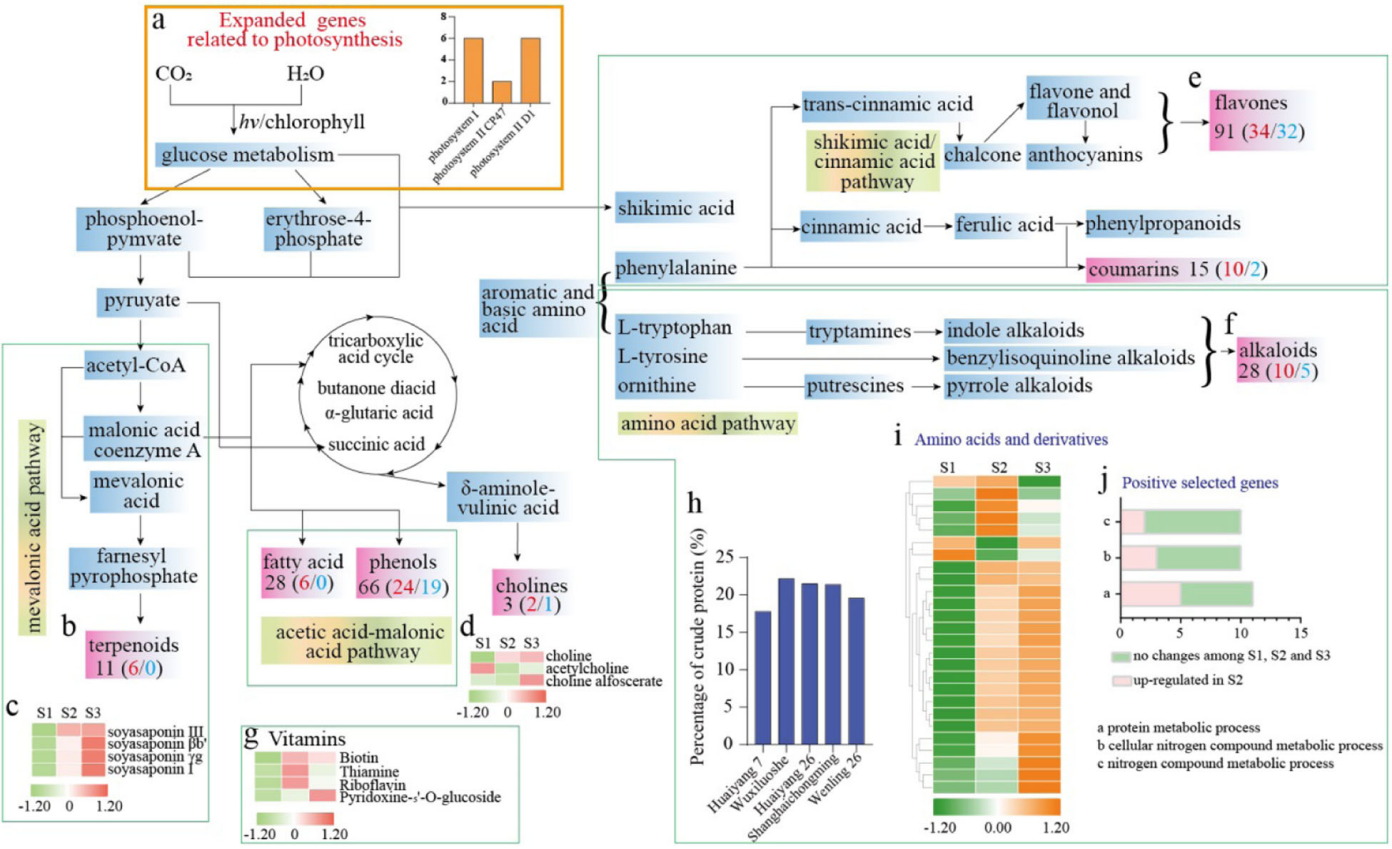
Major nutrient metabolic pathway network. **a** Column diagram showing the number of expanded genes related to photosynthesis. **b** Number of terpenoids. **c** Heatmap showing soyasaponin levels at various growth stages. **d** Heatmap showing choline levels at various growth stages. **e** Number of flavones. **f** Number of alkaloids; dark numbers represent the number of metabolites, and red and blue numbers represent up- and downregulated metabolites from S1 to S3, respectively. **g** Heatmap showing vitamin levels. **h** Percentage of crude protein in various cultivars of *M. polymorpha*. **i** Heatmap showing the levels of amino acids and derivatives and purines. The expression values (FPKM) at the row scale were normalized. Higher mRNA expression levels are indicated in orange, and lower mRNA expression levels are indicated in green. **j** Column diagram showing the number of positively selected genes related to nitrogen metabolism

*M. polymorpha* is a vegetable known for its high levels of nutrients. Notably, comparative genomics revealed that 14 expanded genes were significantly enriched in the category of photosynthesis (*p* < 0.05) (Fig. 5a). These 14 genes included six genes encoding a photosystem I protein (P700 chlorophyll A apoprotein), two genes encoding the photosystem II CP47 reaction center protein, and six genes encoding the photosystem II protein D1 (Fig. 5a). These enriched genes may contribute to enhanced photosynthesis, thereby increasing the accumulation of carbohydrates and nutrients in *M. polymorpha*.

The metabolomics results revealed a total of 492 annotated metabolites belonging to nine distinct biochemical groups: flavonoids (107), lipids (67), phenolic acids (66), amino acids and derivatives (59), nucleotides and derivatives (39), alkaloids (28), terpenoids (11), organic acids (37), and other substances (78), including 13 vitamins, 24 saccharides, and alcohols (Fig. S8). Using principal component analysis (PCA) (Fig. S9) and orthogonal projection to latent structure with discriminant analysis (OPLS-DA) (Fig. S10), we separated these metabolites among developmental stages and narrowed them down to 266 distinct metabolites (VIP > 1 and *p* < 0.05) (Table S9).

Most metabolites were visualized in a network (Fig. 5). The first pathway shown is the mevalonic acid pathway, which produces terpenoid compounds. Six terpenoids were significantly upregulated from S1 to S3 (Fig. 5b). In particular, *M. polymorpha* produced four soyasaponins at high levels at stages S2 and S3 (Fig. 5c). The second pathway is the acetic acid-malonate acid pathway, which biosynthesizes fatty acids and phenols. In this pathway, we detected the upregulation of 6 fatty acids and 24 phenols. The third pathway, the acetyl-CoA pathway, also participates in the tricarboxylic acid cycle, eventually forming cholines. Three cholines were detected in *M. polymorpha*, two of which had high levels in S3 (Fig. 5d). The fourth pathway is the shikimic acid/cinnamic acid pathway, in which flavanols, flavonols, and anthocyanins are synthesized. A total of 91 flavones were detected in *M. polymorpha*, 34 of which showed strong accumulation at S2 and S3 (Fig. 5e). The fifth pathway is the amino acid pathway, in which alkaloids are synthesized. Of the 28 alkaloids detected, 10 showed high levels at S2 and S3 (Fig. 5f). In addition, 13 vitamins were present, and 4 of them (vitamin H: biotin; vitamin B1: thiamine; vitamin B2: riboflavin and vitamin B6: pyridoxine-5’-O-glucoside) were upregulated at S2 (Fig. 5g).

### Genetic basis of crude protein biosynthesis in *M. polymorpha*

We tested the crude protein levels of various cultivars of *M. polymorpha* at the early flowering stage (S2). Our results showed that ‘Wuxiluoshe’ had the highest crude protein percentage (22.2%), followed by ‘Huaiyang 26’, ‘Shanghaichongming’ and ‘Wenlin 26’, with 21.5%, 21.4%, and 19.6% crude protein, respectively. The ‘Huaiyang 7’ cultivar had the lowest percentage (17.8%) (Fig. 5h). The protein content can be used to estimate the nitrogen (N) content of feed samples, and the N content is strongly associated with amino acids and derivatives. In this study, the metabolomic results showed that 26 amino acids and derivatives accumulated significantly in S2 or S3 (Fig. 5i). Furthermore, comparative genomics revealed a total of 118 positively selected genes in *M. polymorpha*. GO enrichment results showed that 11 of these genes were involved in protein metabolic processes, 10 were associated with cellular nitrogen compound metabolic processes, and 10 pertained to organic nitrogen compound biosynthetic processes, and among these groups of genes, 5, 3 and 2, respectively, showed higher expression levels in S2 than in S1 (Fig. 5j).

## Discussion

*M. polymorpha* is an economically important plant species that is widely employed as a forage and vegetable. Here, we generated a high-quality genome assembly for *M. polymorpha*. The assembled genome is ~457.53 Mb in length. The contig and scaffold N50 sizes are 11.02 Mb and 57.72 Mb, respectively, indicating excellent continuity. Moreover, most core eukaryotic genes (96.77%) and genes in the BUSCO datasets (91.8%) were identified, indicating high genome integrity. Synteny analysis showed that *M. polymorpha*, *M. sativa* and *M. truncatula* have a strong genomic syntenic relationship. These results indicate that our genome assembly is precise, complete and of high quality. The chromosome-level genome not only provides a fundamental resource for *M. polymorpha* functional genomic studies but also will help to clarify the biological and evolutionary features of *Medicago*.

Previous studies have revealed that lignin content and monomer composition markedly affect forage digestibility. Decreased lignin content can improve digestibility^20^, whereas a high syringyl/guaiacyl (S/G) ratio is often correlated with reduced degradability in forages^21^. In our study, thinner layers of xylem cells and tissues in *M. polymorpha* compared to *M. sativa* were apparent. This difference would contribute to easier chewing (tender stem/stalk/leaves) and better taste. In addition, metabolomic results showed a low S/G ratio at S2, indicating the easy degradability and digestibility of *M. polymorpha*. In dicot species, 11 main enzymes are involved in monolignol biosynthesis from the aromatic amino acid L-Phe^22^. Furthermore, laccase catalyzes the final step of monolignol polymerization; therefore, it is a crucial enzyme in the lignin pathway^13^. Thus, reduced expression of laccase genes would lead to vascular development arrest with a lack of lignification^23^. Here, a total of 65 lignin-biosynthesis-related genes were identified in the *M. polymorpha* genome, which is a smaller number than that found in *G. max* (142), *M. sativa* (77) or *M. truncatula* (77). The number of genes encoding laccases (17) is significantly smaller than that in *G. max* (48) and *M. truncatula* (21), implying reduced polymerization of monolignols. Based on these results, we suggest that contraction of the lignin synthesis-related gene family had a significant effect on lignin synthesis in *M. polymorpha*.

Plants use energy from sunlight through photosynthesis to prepare storage compounds, such as glucose and starch (carbohydrates). Higher rates of photosynthesis lead to higher levels of carbohydrate accumulation^24^. Moreover, some carbohydrates are converted to other nutritive substances, such as fats, oils, proteins and vitamins. In *M. polymorpha*, we found that 14 enriched genes were related to photosynthesis, indicating a high photosynthetic capacity in this species. In addition, we detected large numbers of nutritive metabolites, including 107 flavonoids, 66 phenolic acids, 28 alkaloids, 11 terpenoids and 13 vitamins. Some of the specific metabolites identified are biologically active, such as vitamin B2, which can regulate sugar, fat, and protein metabolism, in addition to its antioxidative and anti-infective activities^25^. Saponins exhibit protective effects through their antibacterial and anticancer properties^26^. In particular, flavonoids are potent antioxidants^27^. Given that many nutritious compounds, such as vitamins, soyasaponins, and flavonoids, were enriched in *M. polymorpha*, these metabolites may contribute strongly to the nutritional value of *M. polymorpha*.

Forage quality is positively associated with protein concentration. When the percentage of crude protein is low, intake by animals and digestibility are reduced^11^. In alfalfa, the crude protein concentration is ~20% of the dry weight^28^. In *M. polymorpha*, the crude protein percentages of different cultivars ranged between 17.8% and 22.2%, similar to that of alfalfa (tetraploid). Therefore, the diploid *Medicago* plant *M. polymorpha* shows a relatively high crude protein content and good digestibility for use as forage. Previous studies have verified that N metabolism-related genes are important in crude protein biosynthesis^29^. In the present study, several positively selected genes were found to be related to N metabolism. Through metabolic analysis, 26 amino acids and their derivatives were found to accumulate significantly at stages S2 and S3, suggesting that these positively selected N metabolism-related genes could increase protein biosynthesis. Therefore, *M. polymorpha* is an excellent forage plant with high crude protein levels that will benefit livestock.

**Fig. 6:**
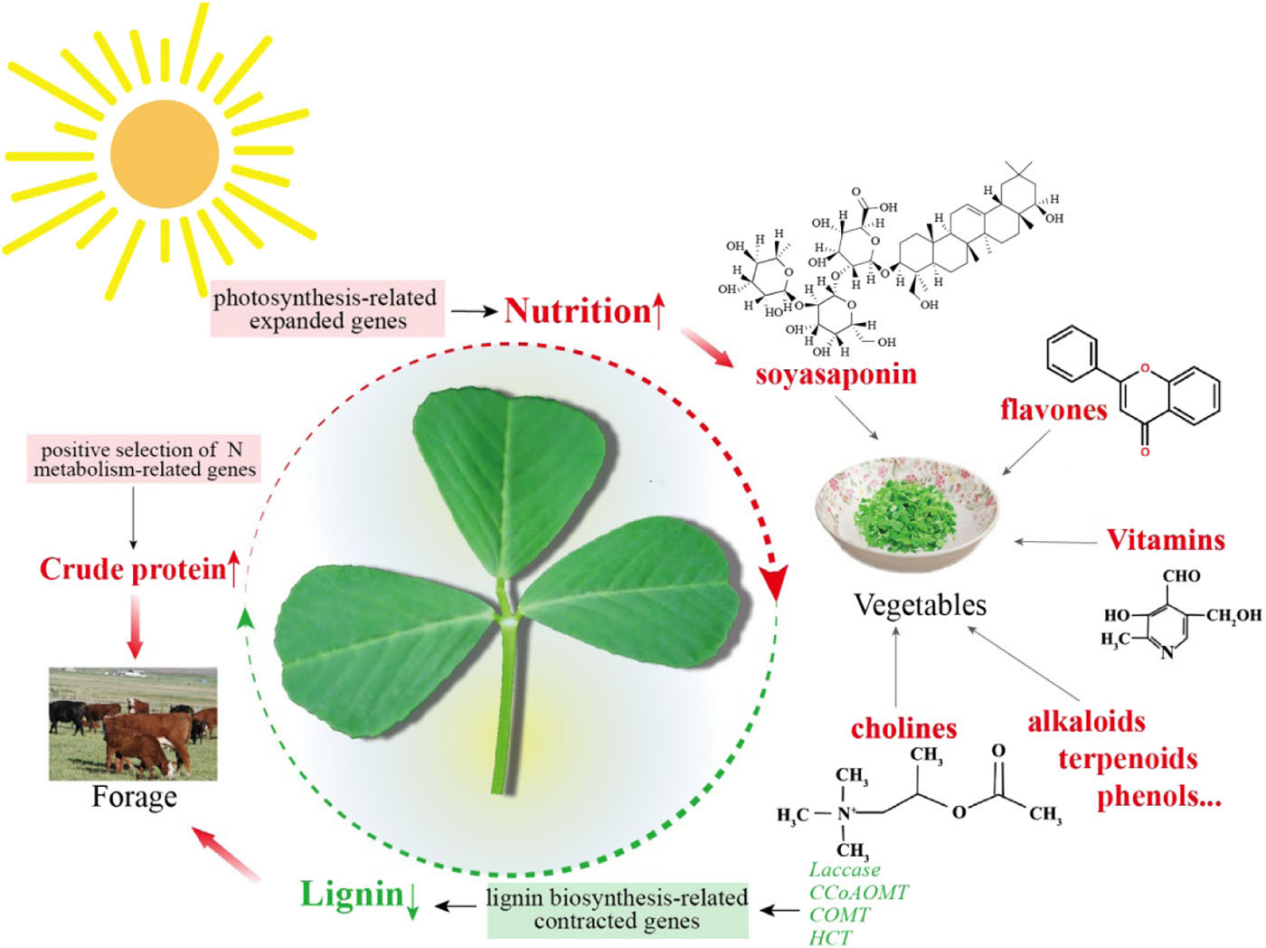
Schematic diagram summarizing the edibility and nutritional value of *M. polymorpha* for both forage and vegetable use. The red and green words represent increased and decreased index values in *M. polymorpha*, respectively

In summary, through analyses of the plant’s edible qualities and nutritional value, this study demonstrated that *M. polymorpha* is an important legume forage for livestock and a nutritious vegetable for human consumption (Fig. 6). The high-quality annotated *M. polymorpha* genome sequence and transcriptomic and metabolomic datasets obtained in this study offer valuable information for both basic research and agronomic improvement of *M. polymorpha* as well as other economically important plants in the genus *Medicago*.

## Materials and methods

### DNA isolation and sequencing

The sequenced specimen of *M. polymorpha* was cultivated at Yangzhou University (32°390′N, 119°420′E) in Yangzhou, China. We isolated genomic DNA from *M. polymorpha* leaves using a plant DNA kit (TIANGEN, Beijing, China). The purified DNA was sequenced using the PacBio RSII (Pacific Biosciences, USA) and HiSeq X Ten (Illumina, USA) sequencing platforms. The clean Illumina genomic reads were used to obtain the k-mer 17 frequency for estimating the genome size of *M. polymorpha* by Jellyfish (v.2.2.9). The overall characteristics of the genome, such as genome size, repeat content and heterozygosity rate, were estimated using GCE software. Low-quality PacBio subreads with a read length shorter than 500 bp or a quality score lower than 0.8 were filtered out. The remaining clean PacBio subreads were error-corrected and assembled into contigs using FALCON software. The assembled scaffolds were polished with Quiver (http://pbsmrtpipe.readthedocs.io/en/master/getting_started.html) in two rounds. Finally, the polished sequences were further corrected with reference to the Illumina reads using Pilon (https://github.com/broadinstitute/pilon/wiki) in two rounds.

### Hi-C library construction and sequencing

Fresh leaves were collected from *M. polymorpha* seedlings for immediate DNA extraction. Following a standard protocol described previously^30^, we constructed Hi-C libraries and conducted sequencing on an Illumina HiSeq X Ten sequencer.

To avoid artificial bias, we filtered the data by removing some types of reads as described previously^31^. The filtered Hi-C reads were aligned to the initial pseudochromosome genome using BWA (version 0.7.8)^32^ with the default parameters. Reads were excluded from subsequent analysis if they did not align within 500 bp of a restriction site. Only uniquely mapped and valid di-tag paired-end reads were used for validation of the pseudochromosome sequences. Juicebox (https://github.com/aidenlab/Juicebox) was used to manually order the scaffolds in each group to obtain the final pseudochromosome assembly. Contact maps were plotted using HICPlotter^33^. High collinearity between the genetic map-based pseudomolecule anchoring and Hi-C-based contact map information corroborated the overall assembly quality (Fig. S2).

### Assessment of genomic integrity

To evaluate the quality of the assembled genome, we used BUSCO (http://busco.ezlab.org/) to examine gene content completeness with the Embryophyta odb9 database and default parameters. Core Eukaryotic Genes Mapping Approach (CEGMA: http://korflab.ucdavis.edu/dataseda/cegma/) was also used to assess assembly quality.

### RNA library construction and sequencing

We performed RNA-seq analysis using the Illumina RNA-seq (separate samples) and PacBio full-length transcript (mixed samples) sequencing methods to improve the predictive ability of gene models. Six different tissues from *M. polymorpha*, including seeds, roots, stems, leaves, flowers, and pods, were collected and frozen in liquid nitrogen. Total RNA was extracted using a Takara Total RNA Extraction Kit (Takara, Dalian, China). Transcriptome libraries were constructed using a NEBNext® Ultra™ RNA Library Prep Kit for Illumina® (NEB, USA) according to the manufacturer’s procedures. Then, 150-bp paired-end sequencing was performed on the Illumina HiSeq X Ten platform. For PacBio sequencing, cDNA was synthesized from the mixed RNA samples used for Illumina sequencing using a SMARTer PCR cDNA Synthesis Kit. After PCR amplification, the products were sequenced on the PacBio RSII platform.

### Genome annotation

Homology alignment and de novo searching were employed to identify repeats. First, homologous repeat sequences were identified based on Repbase. Then, de novo prediction with Repeatmasker was performed.

De novo screening, homology searching, and transcript mapping were used to predict gene models. In homology searching, the *G. max*, *T. subterraneum* and *M. truncatula* protein sequences were aligned to the assembled genome using BLAST (E-value: 1e-5), and gene models were predicted by GeneWise (http://www.ebi.ac.uk/~birney/wise2/). For de novo prediction, gene models were predicted using Augustus (http://bioinf.uni-greifswald.de/augustus/), GlimmerHMM (http://ccb.jhu.edu/software/glimmerhmm/), and SNAP (http://homepage.mac.com/iankorf/). To conduct transcript mapping, transcriptome data from different tissues were aligned to the reference genome using TopHat2. The transcripts were then assembled using StringTie^34^. Finally, EVidenceModeler (EVM, http://evidencemodeler.sourceforge.net/) was used to produce an integrated gene set. Untranslated regions (UTRs) were identified using RNA-seq assemblies.

We predicted the functions of the protein-coding gene set through searches against the SwissProt, Nr, Pfam, KEGG, and InterPro databases.

### Comparative genomic analysis

The genomes of *M. polymorpha* and 14 other plants, including Amborella trichopoda, Oryza sativa, Vitis vinifera, A. thaliana, Populus trichocarpa, M. truncatula, M. sativa, G. max, Cicer arietinum, Cajanus cajan, Trifolium pratense, Vigna angularis, Phaseolus vulgaris, and Arachis duranensis, were used for evolutionary analysis. An all-vs.-all BLASTP (v2.2.28) (E-value: 1e^−5^) search was conducted, and paralogous and orthologous genes were identified by OrthoMCL.

Multiple sequence alignment of all single-copy orthologous genes was performed by MUSCLE (http://www.drive5.com/muscle/). After construction of the integrated supergene sequence based on 4DTv sites, we used RaxML to construct a phylogenetic tree. The divergence times among the 14 species were estimated by MCMCTREE within the PAML package. Calibration points were obtained from the TimeTree database for use as normal priors to confine the ages of nodes.

After gene family cluster analysis, the gene family sizes and the branch lengths of phylogenetic trees were input to CAFÉ (http://sourceforge.net/projects/cafehahnlab/). The expanded or contracted gene families were defined by *p* values smaller than 0.05.

For synteny and whole-genome duplication analysis, MCscanX was used to identify synteny blocks. Then, MUSCLE was used to perform multiple sequence alignment for the sequences of the synteny blocks. Then, the 4DTv value was calculated.

### Copy number analysis of key genes involved in lignin biosynthesis

The protein family from *Arabidopsis* was used as a seed to search for similar protein sequences in *M. polymorpha*, *M. truncatula*, *M. sativa* and *G. max* using the BLAST program in TBtools. Candidates with E-values < 10e^−5^ were then searched against the Pfam 33.1 (http://pfam.xfam.org/) or NCBI batch CDD-search (https://www.ncbi.nlm.nih.gov/Structure/bwrpsb/bwrpsb.cgi) databases to ensure that all major conserved domains present in the seed were also present in the candidates. Candidate protein sequences with very short lengths were manually excluded. The phylogenetic tree was constructed using IQ-TREE (v.1.6.9) by the maximum likelihood method.

### Metabolomic analysis

We collected the aboveground parts of *M. polymorpha* at three different growth stages (S1: seedling stage; S2: early flowering stage; S3: late flowering stage) for metabolomic analysis. First, 100-mg samples were weighed into 2-ml precooled centrifuge tubes. Extraction solvent (0.6 ml of 70% methyl alcohol) was added to the samples, and the mixture was stored at 4 °C overnight with six rounds of vortexing. Then, the samples were centrifuged for 10 min (10,000 rpm). Next, 4 μl of supernatant was injected into a C18 UPLC column (1.8 µm, 2.1 × 100 mm) for ultraperformance liquid chromatography-electrospray ionization-mass spectrometry (UPLC-MS/MS) analysis. For qualitative analysis, we used the MVDB V2.0 database of Wuhan Maiteville Biotechnology Co., Ltd. (Wuhan, China). Metabolites were quantified according to the methods described previously^35^.

## Supplementary information

Supplemental figures

Supplemental tables

## Data Availability

The genome assembly and gene annotation files reported in this paper have been deposited in the Genome Warehouse in National Genomics Data Center, Beijing Institute of Genomics (China National Center for Bioinformation), Chinese Academy of Sciences, under accession number GWHANWO00000000.1 (BioProject: PRJCA003129), which is publicly accessible at https://bigd.big.ac.cn/gwh. The clean DNA-seq data, clean RNA-seq data, and clean Hi-C data of *M. polymorpha* genome have been deposited in the Genome Sequence Archive (GSA) database under accession number CRA003019 (https://bigd.big.ac.cn/gsa/s/q0VtV4XI).
