# Supplementary material for: The genome of Medicago polymorpha provides insights into its edibility and nutritional value as a vegetable and forage legume
Source: Hortic Res. 2021 Mar 1;8:47. doi: 10.1038/s41438-021-00483-5 (PMC7917105; doi:10.1038/s41438-021-00483-5)
Supplement: Supplementary file 1 — Supplemental figures [file 41438_2021_483_MOESM1_ESM.docx]

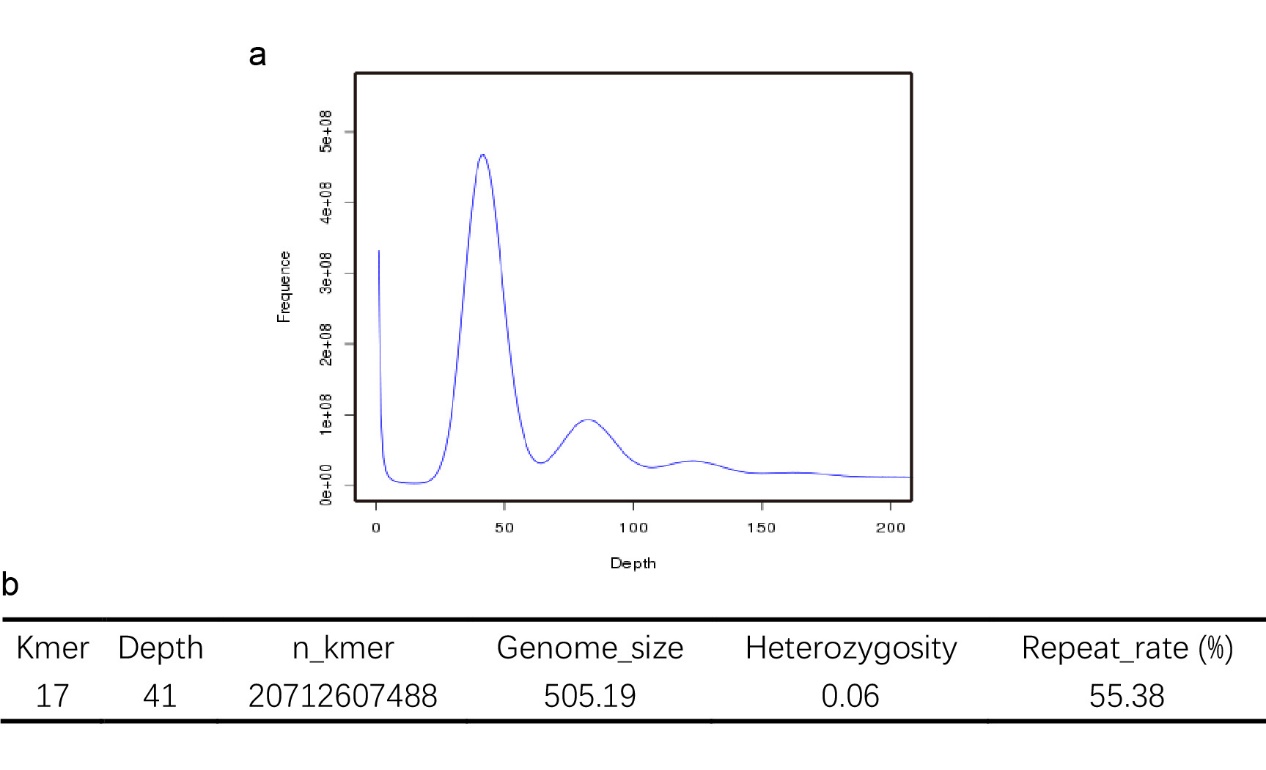


**Fig. S1 Evaluation of *M. polymorpha* genome size by k-mer analysis. a** 17-mer distribution curve. The X-axis is the 17-mer coverage depth, and the y-axis is the k-mer frequency of each k-mer coverage depth. **b** 17-mer statistical data, the genome size was calculated with the first peak k-mer depth (20,712,607,488/41 = 505,185,548).


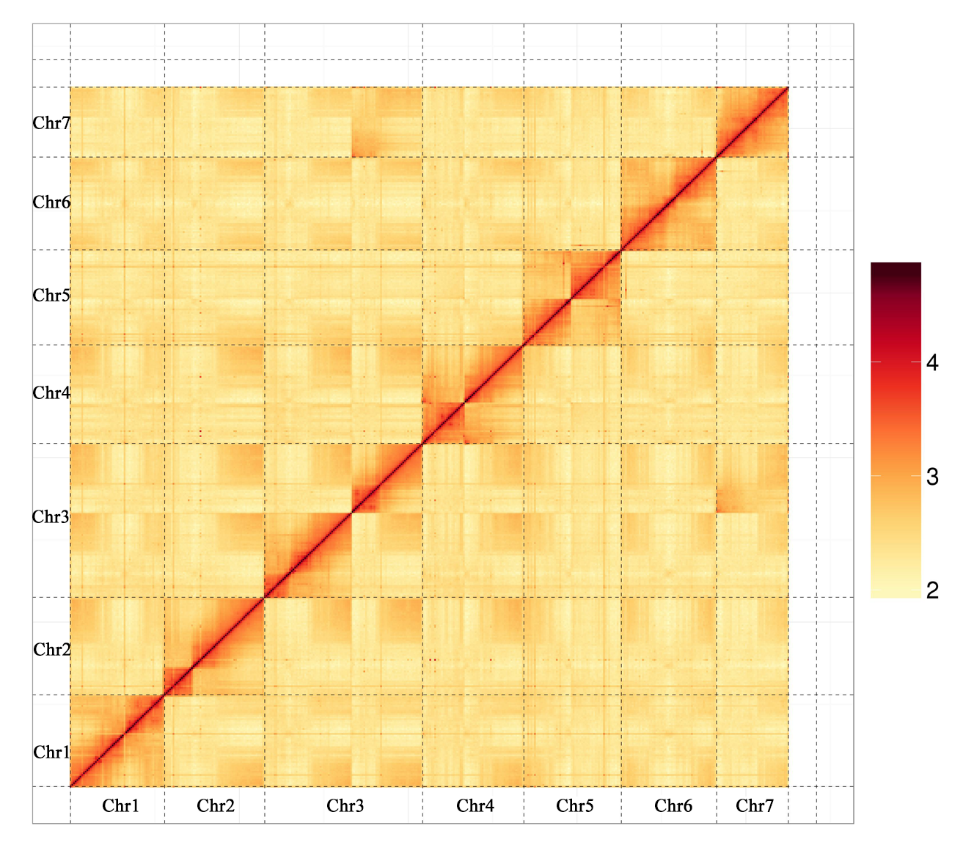


**Fig. S2 Hi-C interaction heatmap of the *M. polymorpha* reference genome showing interactions among the seven chromosomes.** Hi-C reads were realigned to the *M. polymorpha* assembled genome. The intensity of pixels represents the count of Hi-C links, indicating the likelihood of those loci collocating in the nucleus. A darker red color indicates a higher probability of contact.


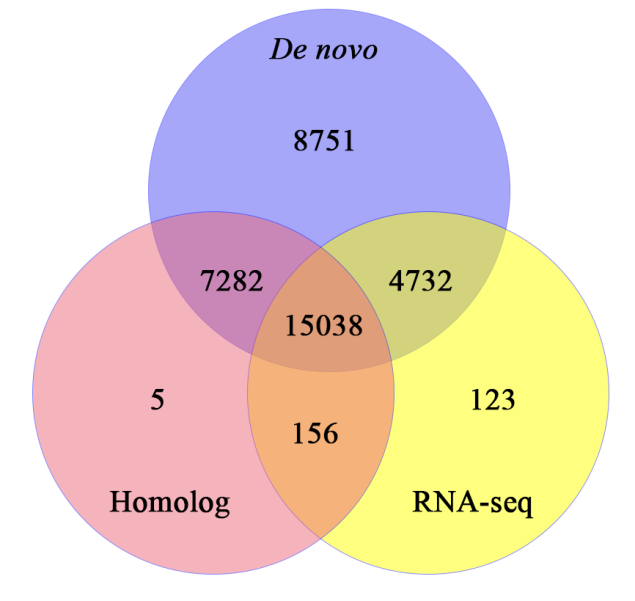


**Fig. S3 Summary of protein-coding genes in the *M. polymorpha* genome predicted using *de novo*, homology-based, and RNA-seq approaches.** All gene models predicted with those three approaches were combined using EVM into a nonredundant set of gene structures. Low-quality gene models were filtered out using the criterion of > 50% gene overlap.


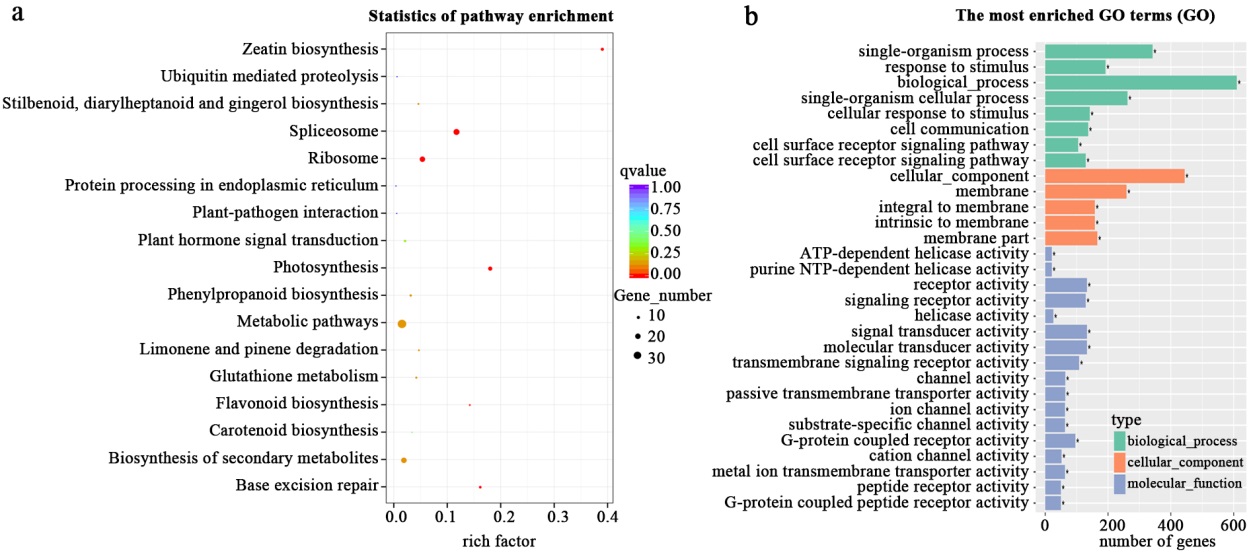


**Fig. S4 Functional enrichment of the expanded gene families in *M. polymorpha*.** **a** Statistics for the top 20 enriched pathways among the expanded gene families. The degree of KEGG enrichment was determined by the enrichment factor, q-value, and gene number. The sizes and colors of spots represent the number of genes and the q-value. **b** GO enrichment analysis of expanded gene families. Green bars represent biological processes, orange bars represent cellular components and blue bars represent molecular functions. * represents significantly enriched GO terms.


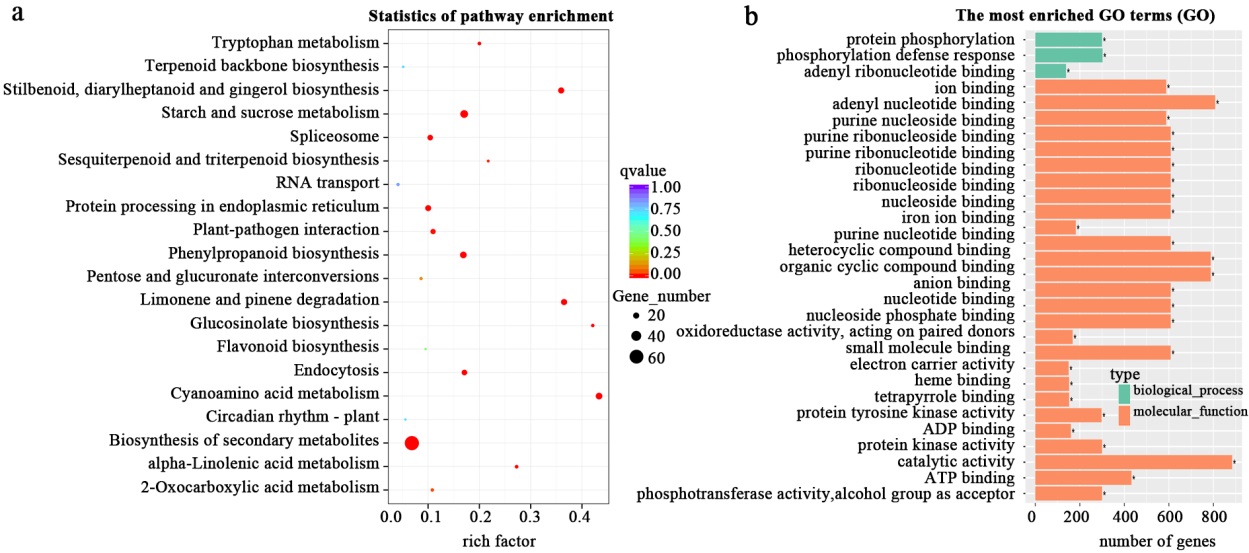


**Fig. S5 Functional enrichment of the contracted gene families in *M. polymorpha*.** **a** Statistics for the top 20 enriched pathways among the contracted gene families. The degree of KEGG enrichment was determined by the enrichment factor, q-value, and gene number. The sizes and colors of spots represent the number of genes and the q-value. **b** GO enrichment analysis of contracted gene families. Green bars represent biological processes, and orange bars represent cellular components. * represents significantly enriched GO terms.


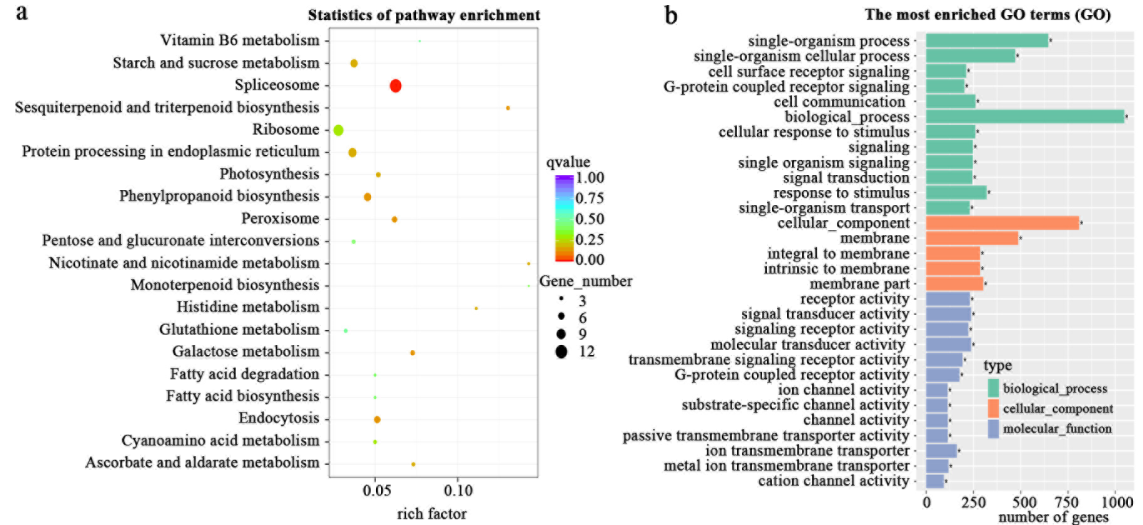


**Fig. S6 Functional enrichment of the *M. polymorpha*-specific gene families.** **a** Statistics for the top 20 enriched pathways among the specific gene families. The degree of KEGG enrichment was determined by the enrichment factor, q-value, and gene number. The sizes and colors of spots represent the number of genes and the q-value. **b** GO enrichment analysis of specific gene families. Green bars represent biological processes, orange bars represent cellular components and blue bars represent molecular functions. * represents significantly enriched GO terms.


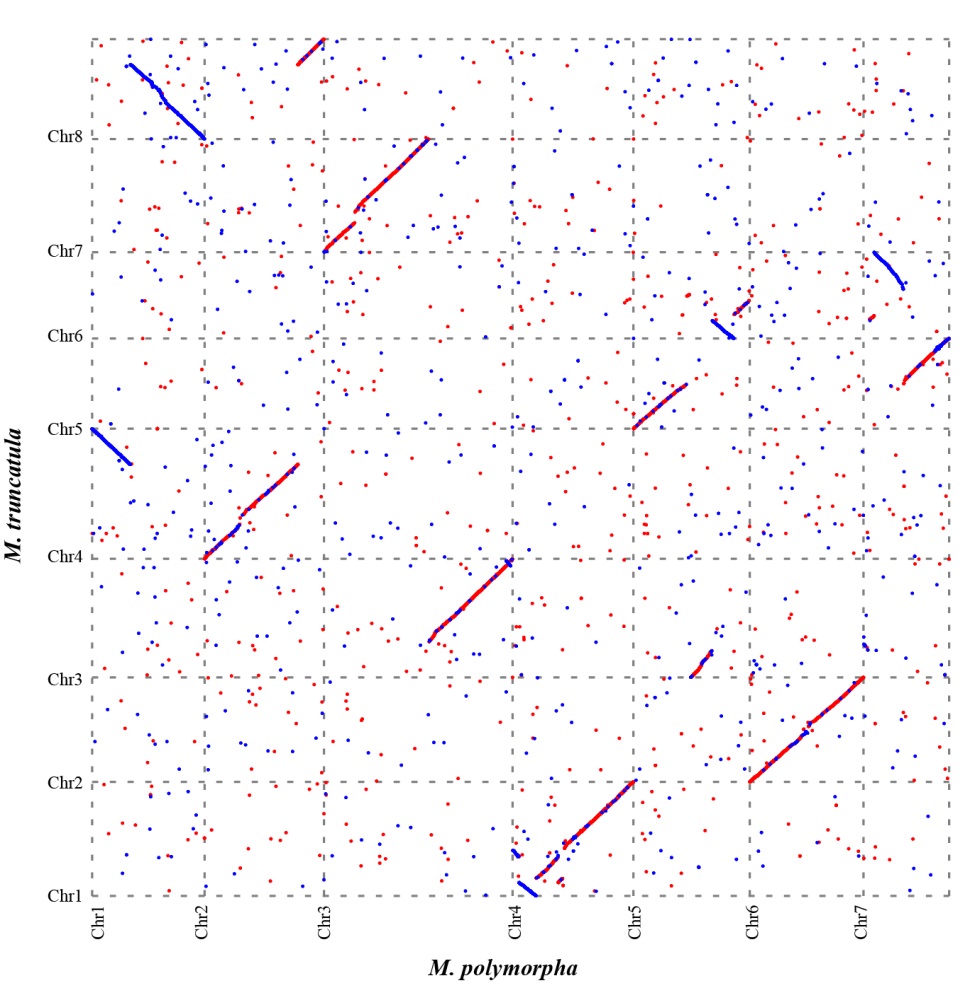


**Fig. S7** **Collinear dot plots for genome comparisons between *M. polymorpha* and *M. truncatula*.** Note that red dots denote positive alignments, while blue dots denote negative alignments. Collinear dot plots were analyzed using mummer (version 4.0).


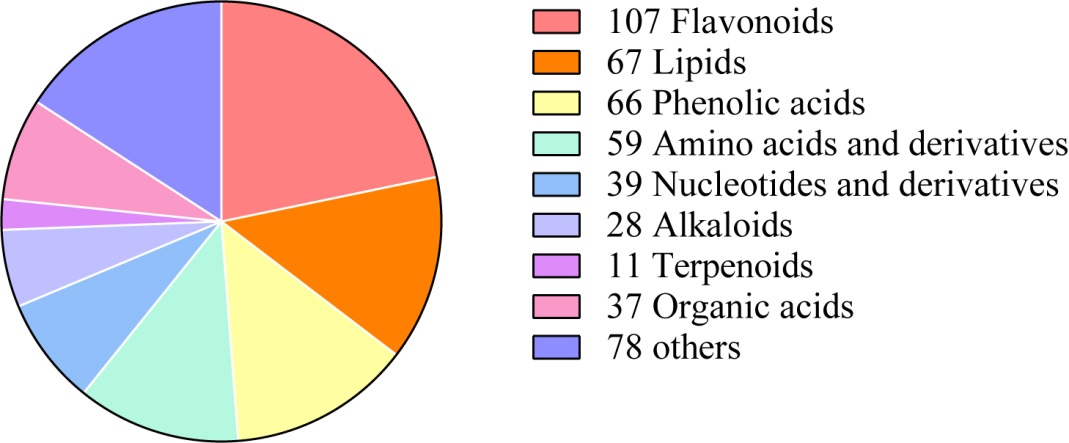


**Fig. S8 Metabolites detected in *M. polymorpha*.** The metabolites were grouped into nine categories, including flavonoids, lipids, phenolic acids, amino acids and derivatives, nucleotides and derivatives, alkaloids, terpenoids, organic acids and others.


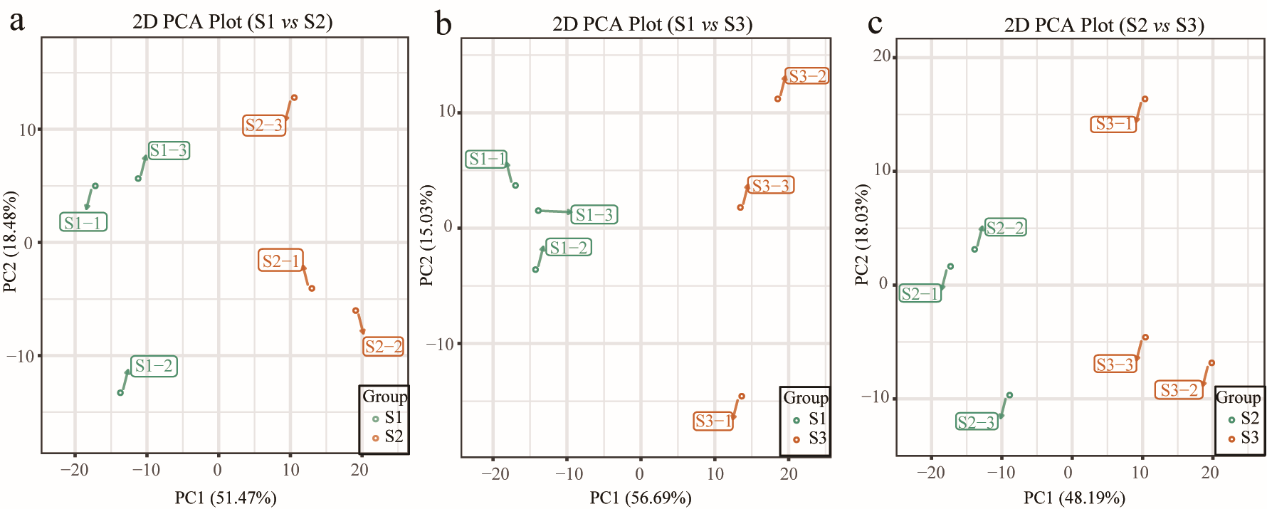


**Fig. S9 Principal component analysis (PCA) of metabolomic results.** **a** S1 vs S2, green circle: S1, orange circle: S2; **b** S1 vs S3, green circle: S1, orange circle: S3; **c** S2 vs S3, green circle: S2, orange circle: S3. Each growth stage contained three biological repeats.


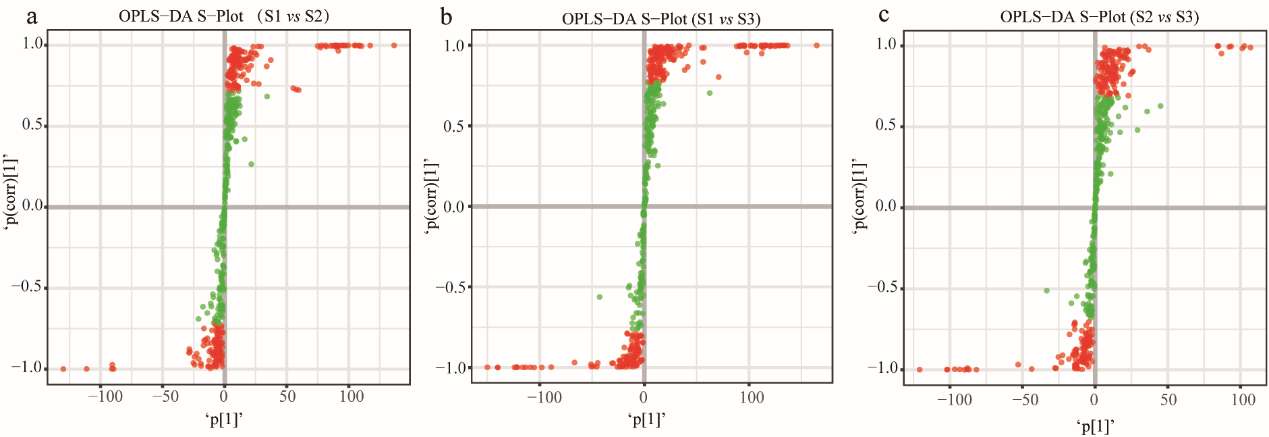


**Fig. S10** **Orthogonal** **projection to latent structure with discriminant analysis (OPLS-DA) of metabolomic results.** The metabolites at different growth stages were analyzed using an OPLS-DA S-plot. a S1 vs S2; b S1 vs S3; c S2 vs S3. The VIPs of metabolites marked in red were ≥ 1, and the VIPs of metabolites marked in green were < 1.
